# Balanced Cultural Identities Promote Cognitive Flexibility among Immigrant Children

**DOI:** 10.3389/fpsyg.2017.01579

**Published:** 2017-09-19

**Authors:** Olivia Spiegler, Birgit Leyendecker

**Affiliations:** ^1^Department of Psychological Methods and Evaluation, University of Hagen Hagen, Germany; ^2^Department of Developmental Psychology, Ruhr University Bochum Bochum, Germany

**Keywords:** biculturalism, identity acculturation, dual identity, ethnic identity, executive functioning, cognitive control

## Abstract

The acculturation complexity model suggests that immersion into dissonant cultures promotes cognitive skills in biculturals (Tadmor and Tetlock, 2006). In the present study, we examined links between identity acculturation and executive functioning (EF). Turkish-German immigrant origin children (*N* = 225; *M* = 11 years, *SD* = 1.6 years, 99 males) were given questions about their identification with Turks and Germans to capture bicultural involvement and a Dot Task (using Hearts and Flowers) to measure EF. Results showed that Turkish-German bicultural children who endorse both cultures with equal strength did not have a cognitive advantage in working memory and inhibition compared to their peers who more clearly preferred one culture over the other. However, bicultural children who endorse both cultures with equal strength performed significantly better on a switching task that required cognitive flexibility. The study highlights the potential cognitive benefits associated with biculturalism.

## Introduction

An increasingly diverse and mobile world has led to a greater interest in the cognitive advantages of biculturalism (Benet-Martínez et al., 2006; Tadmor et al., 2009) which broadly refers to the internalization of two cultures (Nguyen and Benet-Martínez, 2007). The frequent exposure to different cultural values and expectations shapes how biculturals process and react to everyday situations. Immigrants, for example, learn to switch from one language to another and between different cultural meaning systems when interacting with members of the heritage culture versus host culture group (Hong et al., 2000). However, immigrants may relate to their cultural groups in different ways. While some endorse both cultures, others may favor one over the other (Berry, 1997).

The acculturation complexity model (Tadmor and Tetlock, 2006) provides a theoretical link between these varying levels of engagement with two cultures and the development of cognitive skills. The model suggests that biculturals with a relatively equal preference for two distinct cultures experience cultural conflicts and a need to resolve these conflicts. Repeated attempts to resolve cultural discrepancies gradually promote integrative complexity, the cognitive ability to acknowledge, accept, and integrate competing perspectives on the same issue. In contrast, individuals who more clearly prefer one culture over the other are less likely to engage in such complex cognitive processes. For example, Chinese-American adults who internalized both cultures described Chinese and American culture in more complex ways than monoculturals and Chinese-Americans who emphasized one culture (Benet-Martínez et al., 2006). It further seems that the cognitive advantage of biculturals who endorse both cultures with equal strength is not necessarily limited to culture specific tasks but extends to creativity (Gocłowska and Crisp, 2014) and more complex thinking in non-cultural domains (Tadmor et al., 2009).

The present study explores if biculturalism as a form of equal involvement with two cultures also benefits executive functioning (EF). EF covers a set of both unique and overlapping cognitive processes which are required for problem solving and goal achievement (Miyake et al., 2000; Diamond et al., 2007). The core components of EF are *working memory* (mentally holding and using information), *inhibitory control* (deliberate, controlled suppression of prepotent responses), and *cognitive flexibility* (adjusting to change and shifting of mental sets). At present, there is no research on biculturalism (e.g., identity acculturation) and EF, but past research shows that bilingual children are more advanced than their monolingual peers in tasks that require EF components (Adesope et al., 2010; Barac et al., 2014). Both bilingualism, and biculturalism require the ability to effectively alternate the use of culturally appropriate behaviors (LaFromboise et al., 1993). Therefore, we assume that biculturals who endorse both cultures with equal strength will perform better on EF tasks than individuals who favor one culture over the other. In addition, we expect performance differences to be most pronounced in tasks that require cognitive flexibility as the successful navigation of cultures involves the ability to switch between different mind sets (Hong et al., 2000).

Our focus was on Turkish-German children of immigrant origin. This allowed us to investigate the consequences of immersion into two markedly distant cultures in terms of interdependence—independence, family obligations, religious values and traditions, and gender role expectations. The acculturation complexity model argues that the immersion into conflicting and dissonant cultures is most likely to promote cognitive skills (Tadmor and Tetlock, 2006). Our focus on children is of immediate societal relevance as Turkish-German children are the largest immigrant-origin group in Germany and among the least successful in the German school system compared to native Germans and other immigrant groups, and because EF performance is strongly correlated with academic achievement (Best et al., 2011).

## Materials and Methods

### Sample and Procedure

Participants included 225 children of Turkish origin in Germany (*M* = 11 years, *SD* = 1.6, range 9–15 years; 99 male) who attended either fourth (*n* = 147) or seventh (*n* = 78) grade. The sample is drawn from a larger study that investigated resilience processes across countries during school transition periods, hence the two age groups (Leyendecker et al., 2016). The great majority of children were second generation immigrants, which means that they were born in Germany and one (*n* = 55) or both of their parents (*n* = 131) were born in Turkey. Children came from low income families; 73% with a monthly net income below 1,000 Euro. Sampling took place in the Ruhr area, an industrial area in the Northwest of Germany where immigrants make up 25% of the population. Data were collected in the participants’ homes by trained staff and if possible in the child’s room. Parents and siblings were asked to avoid interruptions especially during the EF tasks. Confidentiality was explained, and one of the parents as well as the child signed consent forms. Families received €25 compensation.

### Measures

#### Biculturalism

Heritage and host culture identities are strong indicators of cultural immersion which is why we focused on children’s identification with Turks and Germans as indicators of biculturalism. Among seventh graders Turkish and German identity was measured with six items from the MEIM-R (Phinney and Ong, 2007); e.g., “I have a strong sense of belonging to Turkish/German people.” The MEIM is not considered age appropriate for fourth graders (Reese et al., 1998; Holmes and Lochman, 2009), so we designed six parallel items that also capture children’s positive evaluations of their cultural group membership, a key component of ethnic-racial identity (Ashmore et al., 2004; Rivas-Drake et al., 2014). The items were: “I am happy to be a Turkish/German child,” “Being Turkish/German is an important part of who I am,” and “I feel I belong to Turkish/German children.” Items were scored on a 5-point scale ranging from 1 (*totally disagree*) to 5 (*totally agree*). Factor analyses supported the underlying two-factor structure in each cohort and construct reliabilities ranged from 0.82 to 0.89.

#### Executive Functioning

A Dot Task (using Hearts and Flowers) was administered to measure EF (Diamond et al., 2007). There are three conditions that assess working memory, inhibitory control and cognitive flexibility. On each trial, a red heart or flower appeared for 750 ms on the right or left side of the screen. In the first block, the congruent condition (12 trials) children were asked to “press on the same side as the heart” which required remembering a rule. In the second block, the incongruent condition (12 trials) children were asked to “press on the side opposite the flower.” Incongruent trials require remembering a rule and the suppression of a prepotent response that is the tendency to respond on the side where the stimulus appeared. In the third block, the mixed condition (32 trials), incongruent and congruent trials were intermixed. Therefore, mixed trials require not only memory and inhibition but also cognitive flexibility. Responses faster than 200 ms were excluded from the analyses (Davidson et al., 2006). We report on accuracy rather than reaction times as accuracy is the more sensitive measure for task performance among young children (Diamond and Kirkham, 2005).

## Results

### Biculturalism

On average, children identified more strongly with Turks (*M* = 4.39, *SD* = 0.69) and less with Germans (*M* = 2.82, *SD* = 1.17, *p* ≤ 0.001).

### Executive Functioning

Results of three paired sample *t*-tests revealed that children’s responses on the Dot Task were most accurate in the congruent trials (*M* = 98.52%, *SD* = 3.89%, range 78–100%), followed by the incongruent trials (*M* = 94.52%, *SD* = 7.81%, range 60–100%), and least accurate in the mixed trials (*M* = 75.63%, *SD* = 14.22%, range 40–100%), *p*s ≤ 0.001. Accuracy in the congruent trials was correlated with accuracy in the incongruent trials (*r* = 0.16, *p* = 0.024) and mixed trials (*r* = 0.31, *p* ≤ 0.001), and accuracy in the incongruent trials correlated with accuracy in the mixed trials (*r* = 0.30, *p* ≤ 0.001).

### Biculturalism and EF

**FIGURE 1:**
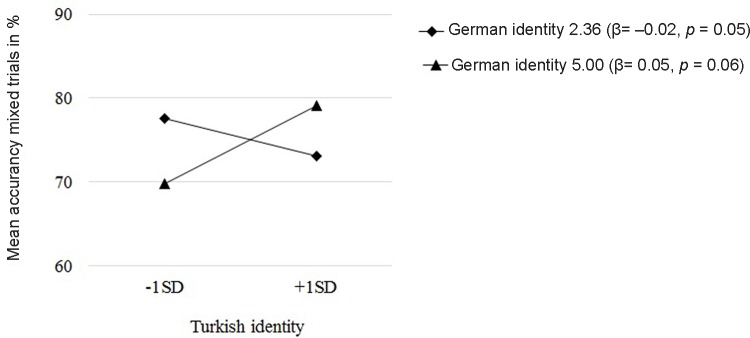
Turkish and German identity interactively predict cognitive flexibility.

To examine links between children’s cultural identities and EF we conducted three independent moderation analyses with Turkish identity, German identity, and Turkish × German identity as predictors and gender, age and family net income as control variables. To get a more precise estimation of the EF components we further controlled for performance in the preceding block(s). All predictor variables were *z*-standardized prior to the analyses. Children’s Turkish and German identity were neither independently nor interactively related to performance in the congruent trials and incongruent trials (**Table 1**). However, children’s Turkish and German identity interactively predicted performance in the mixed trials. The positive interaction coefficient indicated that the relation between Turkish identity and accuracy in the mixed trials was more positive for children with higher levels of German identity. We probed this interaction effect further with the Johnson–Neyman technique (Preacher et al., 2006). This technique defines regions of significance on the moderator for which a simple slope is significantly different from 0. Results indicated that the link between Turkish identity and accuracy in mixed trials was significantly negative (β = -0.02, *p* = 0.05) when German identity was smaller than or equal to 2.36 (regions uncentered lower bound). For children with a German identity at or below that point (40.1%) increasing levels of Turkish identity were significantly linked to poorer performance on mixed trials. The link between Turkish identity and accuracy in mixed trials was marginal significant and positive (β = 0.05, *p* = 0.06) at a German identity level of five (highest possible uncentered value). For these children (5.5%) increasing levels of Turkish identity were linked to better performance in mixed trials (**Figure 1**)^1^.

**Table 1:** Standardized regression coefficients and standard errors of multivariate regression analyses.

	Mean accuracy congruent trial	Mean accuracy incongruent trials	Mean accuracy mixed trials
Intercept	98.54 (0.26)	94.46 (0.56)	75.20 (0.94)
Child gender	0.28 (0.26)	0.69 (0.57)	0.10 (0.96)
Child age	0.52 (0.27)^†^	-0.26 (0.59)	3.73 (0.99)***
Monthly net income family	0.64 (0.26)*	0.01 (0.58)	-0.05 (0.99)
Turkish identity	-0.10 (0.27)	-0.23 (0.60)	-1.01 (1.00)
German identity	-0.07 (0.29)	0.33 (0.63)	-0.42 (1.06)
Turkish × German identity	0.32 (0.33)	0.68 (0.74)	3.05 (1.23)*
Mean accuracy congruent trials	-	2.07 (0.65)**	3.94 (1.12)***
Mean accuracy incongruent trials	-	-	3.43 (1.09)**
Model fit	*F*(6,177) = 1.89^†^	*F*(7,167) = 2.27^∗^	*F*(8,167) = 8.48^∗∗∗^
*R*^2^	0.06	0.09	0.29

*Gender originally coded as 1 (male) and 2 (female). ^†^*p* < 0.10, ^∗^*p* < 0.05, ^∗∗^*p* < 0.01,^∗∗∗^*p* < 0.001.*

## Discussion

The present study is the first to demonstrate that immigrant-origin children who identify equally strong with their ethnic heritage and host-national groups perform better on a switching task that demands cognitive flexibility than their peers who prefer one culture over the other. The immersion into two cultures is highly demanding, especially if both cultures are markedly distinct. Individuals who endorse such distinct cultures with relatively equal strength are expected to switch more often between cultural mindsets and behavioral repertoires than individuals who make a commitment to one of the cultures (Hong et al., 2000). Our results indicate that the more frequent cultural frame switching experiences of biculturals can generalize to greater switching abilities in non-cultural tasks. This complements research on the positive effects of bilingualism and is a valuable contribution to prior research on biculturals’ advanced cognitive complexity.

It is of interest to note that children who reported relatively low identity as both Turkish and German also performed well. Similar findings were reported by Tadmor et al. (2009) who studied cognitive complexity among Asian American college students and Israelis in the United States. They argued that it is the equal preference for two cultures, rather than high levels of identification, that creates dissonance, an internal conflict due to respected, discordant cultural values, and cognitions. Resolving this conflict repeatedly increases cognitive ability.

Biculturalism as a form of equal involvement with two cultures was unrelated to tasks that required inhibitory skills. These results resemble previous findings on bilingual children who do not exhibit any specific advantage in simple inhibitory tasks compared to monolinguals (Duñabeitia et al., 2014) but contradict a large amount of other research on bilinguals’ enhanced ability in inhibiting irrelevant information (Barac et al., 2014). At best, one might argue that culture is less obvious and more subtle than language. Therefore, the need to inhibit culturally specific responses during daily interactions is less strong than the need to suppress unfitting vocabulary. However, this was the first study that investigated EF processes in relation to biculturalism. Moreover, children performed much more accurately in the congruent and incongruent trials compared to the mixed trials indicating a possible ceiling effect. These initial results should therefore be replicated with different measures, larger samples, and using other age groups before drawing premature conclusions.

Arguably our participants were all bilingual to some degree as most of the children had at least one parent who was a first-generation immigrant and all children attended a German school. This provides some support for the unique role of biculturalism in EF development. Actual data on children’s language skills were only available for the seventh graders who reported on their ability to speak, understand, read, and write in both languages. To support our argument that the findings of the study were attributable to identities rather than language skills we tested (for seventh graders only) if Turkish and German identities would also interactively predict accuracy in the mixed trials when Turkish and German language abilities were added as control variables. This was the case but only at a marginal significant level, β = 0.03, *p* = 0.081, *F*(10, 51) = 2.75, *p* = 0.009, *R*^2^ = 0.35. In a next step, we tested if self-reported Turkish and German language abilities interactively predicted accuracy in the mixed trials when controlling for Turkish and German identity, which was not the case β = 0.00, *p* = 0.955. Taken together, these findings support the role of biculturalism rather than bilingualism for performance on a switching task. However, these *post hoc* analyses were based on self-reports of language ability, they were highly underpowered and focused only on a narrow age range which is why future studies need to disentangle the unique effects of biculturalism and bilingualism.

A major limitation of the present study is its correlational design, which does not allow for causal interpretation. It may be that it is easier for children with greater cognitive skills to integrate their multiple memberships of distinct cultural groups, while children with less capacity prefer to endorse one culture. It is also possible that biculturalism and cognitive development mutually influence each other over time. For a more detailed discussion of causality, see Tadmor et al. (2009). Either way, it is the link between the two concepts that is most relevant for intervention programs. On the one hand, Turkish immigrant children seem to need an open space in which they can get involved with people and values from both cultural groups to ultimately succeed in the educational domain. On the other hand, school programs that improve EF and, specifically, cognitive flexibility (Diamond et al., 2007) may have positive side-effects for Turkish immigrant children’s integration.

What is it like to grow up bicultural? It seems that a significant part of the bicultural experience is switching between different cultural frames of reference. Some may find this bothersome and stressful whereas others are intrigued and challenged. There may be a chance that the latter will be rewarded with greater cognitive flexibility, a skill that enables one to adjust quickly and to consider things from fresh and different perspectives, a vital skill for adaptation and creativity.

## Ethics Statement

This study was carried out in accordance with the recommendations of the ethics committee of the German Psychological Society with written informed consent from all subjects. All subjects gave written informed consent in accordance with the Declaration of Helsinki. The protocol was approved by the ethics committee of the German Psychological Society (DGPs, Letter of approval from May 7, 2010).

## Author Contributions

OS analyzed data and drafted this work. BL revised the manuscript. All else shared.

## Conflict of Interest Statement

The authors declare that the research was conducted in the absence of any commercial or financial relationships that could be construed as a potential conflict of interest.

## Footnotes

1 We explored if children’s age moderated the link between biculturalism and accuracy in the mixed trials by conducting a multivariate regression analysis with a three-way interaction term (Turkish identity × German identity × age). This interaction was not significant, β = 0.01, *SE* = 0.01, *p* = 0.643, indicating that the link between biculturalism and cognitive flexibility did not depend on children’s age.
